# Proteomic Signature of Dysfunctional Circulating Endothelial Colony‐Forming Cells of Young Adults

**DOI:** 10.1161/JAHA.121.021119

**Published:** 2021-07-19

**Authors:** Cheryl M. J. Tan, Adam J. Lewandowski, Wilby Williamson, Odaro J. Huckstep, Grace Z. Yu, Roman Fischer, Jillian N. Simon, Maryam Alsharqi, Afifah Mohamed, Paul Leeson, Mariane Bertagnolli

**Affiliations:** ^1^ Oxford Cardiovascular Clinical Research Facility, Radcliffe Department of Medicine, Division of Cardiovascular Medicine University of Oxford Oxford UK; ^2^ Department of Biology United States Air Force Academy Colorado Springs CO USA; ^3^ Wellcome Centre for Human Genetics University of Oxford Oxford UK; ^4^ Target Discovery Institute (TDI) Mass Spectrometry Laboratory, Target Discovery Institute, Nuffield Department of Medicine University of Oxford Oxford UK; ^5^ Division of Cardiovascular Medicine, Radcliffe Department of Medicine University of Oxford Oxford United Kingdom; ^6^ Department of Cardiac Technology Imam Abdulrahman Bin Faisal University Dammam Saudi Arabia; ^7^ Department of Diagnostic Imaging & Applied Health Sciences, Faculty of Health Sciences Universiti Kebangsaan Malaysia Kuala Lumpur Malaysia; ^8^ Montreal Hospital Sacré‐Cœur Research Centre Centre Intégré Universitaire de Santé et de Services Sociaux du Nord‐de‐l'Île‐de‐Montréal Montréal QC Canada; ^9^ School of Physical and Occupational Therapy, Faculty of Medicine McGill University Montréal QC Canada

**Keywords:** angiogenesis, blood pressure, cardiovascular disease risk factors, endothelial progenitor cells, hypertension/high blood pressure, proteomics, Basic Science Research, Angiogenesis, Proteomics, Stem Cells, Vascular Biology

## Abstract

**Background:**

A subpopulation of endothelial progenitor cells called endothelial colony‐forming cells (ECFCs) may offer a platform for cellular assessment in clinical studies because of their remarkable angiogenic and expansion potentials in vitro. Despite endothelial cell function being influenced by cardiovascular risk factors, no studies have yet provided a comprehensive proteomic profile to distinguish functional (ie, more angiogenic and expansive cells) versus dysfunctional circulating ECFCs of young adults. The aim of this study was to provide a detailed proteomic comparison between functional and dysfunctional ECFCs.

**Methods and Results:**

Peripheral blood ECFCs were isolated from 11 subjects (45% men, aged 27±5 years) using Ficoll density gradient centrifugation. ECFCs expressed endothelial and progenitor surface markers and displayed cobblestone‐patterned morphology with clonal and angiogenic capacities in vitro. ECFCs were deemed dysfunctional if <1 closed tube formed during the in vitro tube formation assay and proliferation rate was <20%. Hierarchical functional clustering revealed distinct ECFC proteomic signatures between functional and dysfunctional ECFCs with changes in cellular mechanisms involved in exocytosis, vesicle transport, extracellular matrix organization, cell metabolism, and apoptosis. Targeted antiangiogenic proteins in dysfunctional ECFCs included SPARC (secreted protein acidic and rich in cysteine), CD36 (cluster of differentiation 36), LUM (lumican), and PTX3 (pentraxin‐related protein PYX3).

**Conclusions:**

Circulating ECFCs with impaired angiogenesis and expansion capacities have a distinct proteomic profile and significant phenotype changes compared with highly angiogenic endothelial cells. Impaired angiogenesis in dysfunctional ECFCs may underlie the link between endothelial dysfunction and cardiovascular disease risks in young adults.

**Nonstandard Abbreviations and Acronyms**
AcLDL: acetylated low‐density lipoprotein
ECFC: endothelial colony‐forming cell
ECSCR: endothelial cell‐specific chemotaxis receptor
ENG: endoglin
eNOS: endothelial nitric oxide synthase
EPC: endothelial progenitor cell
ERG: ETS‐related gene
HUVEC: human umbilical vein endothelial cell
LUM: lumican
PBMC: peripheral blood mononucleated cell
PECAM‐1: platelet endothelial cell adhesion molecule‐1
PTX3: pentraxin‐related protein PYX3
RT: room temperature
SPARC: secreted protein acidic and rich in cysteine
UEA‐1: ulex europaeus agglutinin I
VE‐cadherin: vascular endothelial cadherin
VEGFR‐2: vascular endothelial growth factor‐2
vWF: von Willebrand factor

**Clinical Perspective**

**What Is New?**

- We identified a distinct proteomic signature of dysfunctional endothelial colony‐forming cells (ECFCs) with impaired angiogenesis and expansion capacities. Functional ECFCs with higher angiogenic capacity exhibit a greater proteome similarity with human umbilical vein endothelial cells than dysfunctional ECFCs.
- Subjects with dysfunctional circulating ECFCs have higher blood pressure, body mass index, and impaired lipid profile in comparison with subjects with functional ECFCs.
- Proteins of cellular mechanisms involved in exocytosis, vesicle transport, extracellular matrix organization, cell metabolism, and apoptosis were differently expressed in dysfunctional ECFCs in comparison with highly angiogenic endothelial cells.

**What Are the Clinical Implications?**

- Dysfunctional ECFCs with a significant impairment of angiogenesis and expansion capacities may underlie the link between endothelial dysfunction and cardiovascular disease risks in young adults.

Strategically embedded between the tissues and blood, the vascular endothelium constitutes a highly heterogenic, complex, and dynamic structure regulating important physiological processes such as inflammation, vasomotor responses, mechano‐sensing angiogenesis, and hemostasis.^1^, ^2^, ^3^ Hence, impairment of the endothelial cells and vascular endothelium can precede the initiation and progression of cardiovascular diseases, including hypertension, which can also lead to further endothelial dysfunction.^4^, ^5^, ^6^

The human endothelial colony‐forming cells (ECFCs), a subpopulation of endothelial progenitor cells (EPCs), offer a potential platform for cellular assessment because of their remarkable angiogenic, expansion, regenerative, and self‐renewable potentials in vitro.^7^, ^8^, ^9^, ^10^, ^11^, ^12^, ^13^, ^14^, ^15^ However, the literature still lacks important information on the main mechanisms that intrinsically or extrinsically regulate angiogenesis in these cells. This is important because clinical studies have pointed to the fact that dysfunctional ECFCs, particularly in babies and young adults, may be related to lifelong illnesses.^6^, ^16^, ^17^ In spite of this, the phenotype of dysfunctional ECFCs is not well known. Likely originating in the bone marrow or from vessels and pools of progenitor cells in tissues,^18^ ECFCs can be isolated from relatively low volumes (10–25 mL) of peripheral blood.^7^, ^8^, ^9^ Unlike hematopoietic progenitor cells, ECFCs do not express CD45 (cluster of differentiation 45) and CD133 (cluster of differentiation 133).^19^ They express, however, endothelial surface markers including PECAM‐1 (platelet endothelial cell adhesion molecule‐1 or CD31 [cluster of differentiation 31]), ENG (endoglin or CD105 [cluster of differentiation 105]), MCAM (melanoma cell adhesion molecule or CD146 [cluster of differentiation 146]), eNOS (endothelial nitric oxide synthase), VE‐cadherin (vascular endothelial cadherin or CD144 [cluster of differentiation 144]), in addition to progenitor cell antigens, and are also characterized by the uptake of AcLDL (acetylated low‐density lipoprotein).^19^, ^20^

These readily attainable and proliferative ECFCs, also referred to as late outgrowth endothelial cells^14^ or blood outgrowth endothelial cells,^21^ are phenotypically distinct from early EPCs.^14^, ^22^ Typically, early EPCs form colonies within 4 days after peripheral blood mononucleated cell (PBMC) culture and last no longer than 7 to 9 days in culture on collagen‐I coated surfaces, as compared with 6 to 30 days for ECFC culture.^9^, ^12^, ^13^, ^19^, ^20^, ^23^ Early EPCs also have no expansion potential or standard growth pattern,^12^, ^13^ suggesting a myeloid‐like phenotype. ECFCs, on the other hand, exhibit an endothelial lineage commitment with the expression of genes and proteins involved in vascular development and regeneration.^22^

Despite the low number of ECFCs circulating in the blood, animal and clinical studies have shown that in vitro angiogenic and expansion potential can relate to clinical characteristics, disease risk, and repair capacity.^6^, ^10^, ^11^, ^16^, ^24^, ^25^, ^26^, ^27^, ^28^, ^29^ However, no study has yet provided a comprehensive molecular characterization and description of disrupted mechanisms in less angiogenic ECFCs, also classified as dysfunctional cells. This knowledge is critical to justify their use in clinical investigations and to target the discovery of disease‐related cellular mechanisms. Herein, we aimed at comparing the phenotype and proteomic signatures of functional and dysfunctional circulating ECFCs derived from peripheral blood of young adults with no preexisting cardiovascular diseases.

## Methods

### Data Availability Statement

The raw mass spectrometry proteomics data have been deposited to the ProteomeXchange Consortium (http://www.proteomexchange.org) via the Proteomics Identifications Database partner repository with the data set identifier: P XD020677 and project DOI: 10.6019/PXD020677. Further data that support the findings of this study are available from the corresponding author upon request.

### Study Population

Participants were recruited as part of a baseline assessment visit for a study in the Oxford Cardiovascular Clinical Research Facility. Ethical approval was provided by the Oxford Research Ethics Committee (ethics reference: 16/SC/0016). For this study, we included 11 young adults (5 men and 6 women) aged between 18 and 35 years, body mass index (BMI) <30 kg/m^2^, clinical blood pressure <159 mm Hg systolic and/or <99 mm Hg diastolic without any clinical diagnosis of cardiovascular diseases and medications. All participants provided written consent for the collection and subsequent experimental use of samples in accordance with appropriate ethical approvals.

### Anthropometry and Blood Pressure Measurements

Height and weight were measured to the nearest 0.1 cm and 0.1 kg, respectively, using a Seca measuring station (Seca, Birmingham, UK). Footwear was removed before the measurements. Resting brachial blood pressure was measured after a 5‐minute acclimation period in a seated position using an automated oscillometer device (Dinamap V100; GE Healthcare, Chalfont St. Giles, UK). Three readings were taken from the left arm, with the last 2 readings averaged and subsequently analyzed. Trained investigators and clinical staff collected all measurements.

### Blood Sampling

Participants were instructed to have fasted at least 4 hours before blood sample collection. They were encouraged to drink water during the fast. A total volume of 25 mL of peripheral venous blood was collected per individual. All blood samples were collected from the antecubital fossa via venipuncture. Separated plasma and serum were then pipetted and stored at −80 °C for future analysis. Fasting blood biochemistry was measured at the Oxford John Radcliffe Hospital Biochemistry Laboratory using routine validated assays with clinical quality controls. Insulin resistance was calculated using the homeostasis model assessment calculator.^30^

### PBMC Isolation

ECFCs were separated from PBMCs from 15 mL of the collected blood following a protocol adapted from Bertagnolli et al.^6^ First, PBMCs were separated using the Ficoll‐Paque PLUS (GE Healthcare Life Sciences, Pittsburgh, PA) density gradient following 30 minutes and 300*g* centrifugation at room temperature, and then washed twice with Dulbecco's PBS (Gibco by Life Technologies).

### ECFC Isolation and Culture

The process of ECFC isolation from PBMCs is illustrated in Figure S1. PBMCs were plated on collagen I‐coated (Corning, Corning, NY) 25‐cm^2^ tissue culture–treated Falcon flasks (Thermo Fisher Scientific, Waltham, MA) at a density of 5.0×10^6^ cells/flask following standard protocols.^6^, ^31^ Cultured cells were maintained in a standard cell culture condition (humidified chamber, maintained at 37 °C with 21% O_2_ and 5% CO_2_), using complete endothelial cell growth basal medium‐2 (EBM‐2 plus SingleQuots of Growth Supplements; Lonza, Basel, Switzerland) supplemented with 1% penicillin/streptomycin (Gibco by Life Technologies) and 10% FBS (Invitrogen). Media were changed every 2 or 3 days, and cells were maintained for up to 30 days in culture. PBMC cultures were observed daily from days 6 to 30 to determine the first day of cobblestone patterned ECFC colony formation (Figure S1). Once ECFC colonies were identified, they were expanded for no longer than 10 days to control the time of colony formation. These cells were then passaged and plated in similar conditions for all individuals (ie, same surface area in collagen‐coated flasks and equal cell density). ECFC colonies were then passaged and further expanded under similar conditions. ECFC function was assessed using cells from the second passage.

### Human Umbilical Vein Endothelial Cell Culture

Four samples of primary human umbilical vein endothelial cells (HUVECs) derived from healthy pregnancies were obtained from Oxford Cardiovascular Tissue Bioresource of umbilical‐derived cell^32^ (ethics reference: 09/H0606/68, 07/H0606/148, 15/SC/0027, 11/SC/0230). HUVECs were cultured in the same media and standard conditions as ECFCs. HUVEC function was assessed using cells after the second passage.

### Primary Human Dermal Fibroblast

Normal human dermal fibroblasts (Lonza; lot no.: 0000520141) were cultured in fibroblast growth medium‐2 supplemented with FGM‐2 SingleQuots supplements (Lonza). These cells were used as an immunofluorescence negative staining control.

### Fluorescence‐Activated Cell Sorting

ECFCs were detached with Accutase (STEMCELL Technologies, Vancouver, Canada) and washed with PBS. Cells were fixed and permeabilized with 4% paraformaldehyde and 0.2% Triton X‐100, respectively (10 minutes each). Cells were then blocked with 3% FBS for 15 minutes at room temperature (RT). Cells were incubated with conjugated antibodies for 30 minutes at RT (Table S1). Stained and unstained cells were washed with eBioscience flow cytometry staining buffer and analyzed on a Fortessa flow cytometer. Cells were stained with fluorescein isothiocyanate‐PECAM‐1, phycoerythrin‐VE‐cadherin, and allophycocyanin‐VEGFR‐2 (vascular endothelial growth factor‐2), as detailed in Table S1. The fluorescence minus one control is provided in Figure S2.

### Proliferation Assay

The proliferation rate was assessed through the quantification of 5‐ethynyl‐2′‐deoxyuridine (EdU) cellular incorporation using the Click‐iTTM EdU Alexa FluorTM 488 Imaging kit (Thermo Fisher Scientific). There were 4.0×10^4^ ECFCs plated on collagen‐coated flasks, 1.86‐cm^2^ surface area, maintained with complete EBM‐2 media, and grown for 24 hours. Similar culture conditions were used for all individuals. Cells were then incubated with 10 µmol/L EdU in complete EBM‐2 media for 4.5 hours at 37 °C, then fixed with 3.7% formaldehyde in PBS, permeabilized with 0.5% Triton X‐100 in PBS, and stained according to kit instructions. Assays were performed in triplicate and cell images obtained using a fluorescence microscope (Leica, Wetzlar, Germany) and a ×10 objective. The number of cells under proliferation and incorporating EdU into their DNA, stained positive for EdU (AlexaFluor 488), were counted in 3 to 5 full pictures per assay, as well as the total number of cells with nuclei stained with 4′,6‐diamidino‐2‐phenylindole. The percentage of proliferating cells (a measure of proliferation rate) was then calculated.

### In Vitro Tube Formation Assay

In vitro vascular tube formation was assessed by plating 1.5×10^4^ cells on 50 µL of growth factor reduced basement membrane matrix (Matrigel) in a 96‐well plate. Cells were imaged using a Leica DMIL inverted trinocular phase contrast fluorescence microscope (Leica) and ×5 objective after 6 hours of incubation under standard culture conditions. Assays were performed in triplicate, and the number of closed tubes and branches formed (not necessarily forming closed tubes) were quantified in 3 to 6 random images per participant using ImageJ software (National Institutes of Health, Bethesda, MD).

### ECFC Functionality Classification

Cellular proliferation and angiogenic capacity were assessed to classify the functionality of ECFCs. Specifically, ECFCs with <1 closed tube formed on Matrigel, and <20% proliferation rate was deemed dysfunctional. The 20% proliferation cutoff was established based on the capacity of ECFCs to sufficiently expand in culture to form cobblestone‐shaped colonies after the first or second passages and reach at least 30% confluence. As a result, both functional phenotype and proteomic tests were performed using cells obtained from no more than 2 passages after PBMC culture.

### Immunofluorescence Staining

Antibodies and dilutions used are summarized in the Supplementary Methods. Cells (ECFCs, HUVECs, and fibroblasts) were fixed in 4% paraformaldehyde, blocked with blocking buffer (PBS with 10% FBS; 30 minutes, RT), permeabilized (5 minutes, RT) with 0.2% Triton‐X‐100, and incubated with primary antibodies (overnight, 4 °C). After washing, cells were incubated with an appropriate secondary antibody, and nuclei were stained with TOPRO‐3 Iodide (1:500) (1 hour, RT). Washed cells were mounted in SlowFade Gold antifade mountant (Thermo Fisher Scientific) and visualized using a Leica DM6000 CFS confocal microscope with 3 main fluorochromes, Alexa Fluor 488, Alexa Fluor 555, and Alexa Fluor 647, and 12‐bit resolution images were captured with the Leica application suite (version 2.7.3.9723). Appropriate isotype controls were stained (Figure S3). Antibodies used are listed in Table S1.

### Sample Digestion and Desaltation

HUVECs and ECFCs (total n=12) were used for proteomic analysis at passage number 2. Cell pellets (cell count) were resuspended in lysis buffer (50 mmol/L Tris‐HCl pH 8.5, 4% SDS, and 50 mmol/L dithiothreitol), boiled (5 minutes) and incubated (30 minutes, RT) for full protein solubilization. Total protein (≈250 µg) was digested and desalted after 2 rounds of chloroform‐methanol precipitation reduction/alkylation with dithiothreitol/iodoacetamide.^33^ Digested and desalted proteins were dissolved in 0.1% formic acid and loaded into the nano‐LC‐MS/MS (liquid chromatography with tandem mass spectrometry) system (Orbitrap Fusion Lumos; Thermo Fisher Scientific) in collaboration with the Oxford Target Discovery Institute.

### Mass Spectrometry Report System and Conditions

Mass spectrometry analysis was performed in the Target Discovery Institute Mass Spectrometry laboratory led by Benedikt M. Kessler (University of Oxford, Oxford, UK). Peptides were separated with a gradient of 2% to 35% acetonitrile in 0.1% formic acid/5% DMSO on an EasySpray column (50 cm×75 µm) over 60 minutes and with a flow rate of 250 nL/min. Mass spectrometry data were acquired on an Orbitrap Fusion Lumos using a standard method, as described earlier.^34^ For quality control, a sample pool was generated and coanalyzed with the sample data. All samples were processed in the same batch.

### Proteomics Data Availability and System Biology Analyses

Peptides and proteins were identified by searching the mass spectrometry raw files against the Human SwissProt database downloaded in November 2015 (containing 20 268 human sequences). Mascot data outputs were filtered by applying a 20‐ion cutoff and 1% false discovery rate above identity or homology threshold. The raw mass spectrometry proteomics data have been deposited to the ProteomeXchange Consortium (http://www.proteomexchange.org) via the Proteomics Identifications Database partner repository with the data set identifier: PXD020677 and project DOI: 10.6019/PXD020677.

Perseus (Max Planck Institute of Biochemistry, Martinsried, Germany) was used for quantitative analysis of the log‐transformed (normalized protein abundance) after label‐free quantitation as performed in Progenesis QI (Waters) using default settings. The data were filtered per row for 70% of the valid values and ≥2 identified peptides. Student *t* tests were used to make comparisons between the groups, considering genes statistically significant when Log_2_(fold change) were ≤−1.5 or ≥1.5 and false discovery rate–corrected *P* value (here named *q*‐value) <0.05. As part of the proteomic analysis, all the *P* values were expressed as false discovery rate–corrected *P*‐value (*q*‐value) unless stated otherwise. Gene ontology enrichment among cluster‐enriched, differential genes were computed and retrieved with the following ontology sources: KEGG Pathway, GO Biological Processes, Reactome Gene Sets, Canonical Pathways, and CORUM using Metascape (http://www.metascape.org).^35^ Functional protein association networks of identified proteins were created using STRING (http://www.string‐db.org) and InstantClue software.^36^

### Statistical Analysis

All statistical analyses were performed using SPSS version 25 (IBM, Armonk, NY). Shapiro‐Wilk normality test was used to assess the normality of variables. Dependent on the normality of sample distribution, comparisons between 2 groups were performed by either parametric independent *t* test or nonparametric Mann‐Whitney *U* test. Kruskal‐Wallis test was performed with Bonferroni post hoc test for comparisons between HUVEC and both ECFC groups (functional and dysfunctional). Categorical variables were compared by χ^2^ or Fisher exact tests. Results are presented as mean±standard deviation unless stated otherwise. Pearson correlations (*r*) were used for bivariate associations. *P*<0.05 were considered statistically significant.

## Results

### ECFC Phenotype

**Figure 1:**
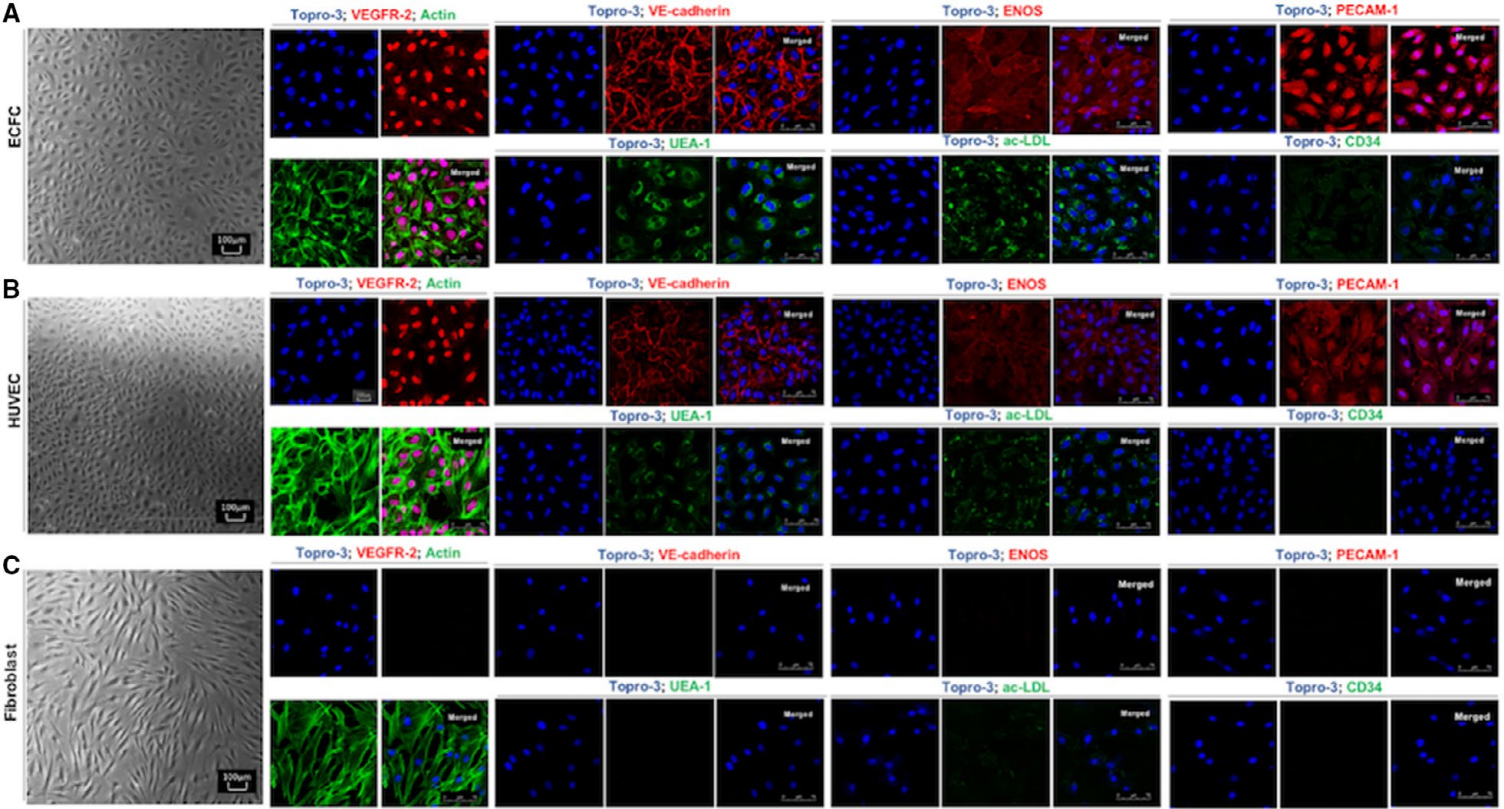
Endothelial colony‐forming cells (ECFCs) phenotype (**A**) in comparison with human umbilical vein endothelial cells (HUVECs) (**B**) and dermal fibroblasts (**C**). HUVECs and fibroblasts served as positive and negative controls. Light microscope images of ECFCs, HUVECs, and fibroblasts. All cells were stained with surface biomarkers including VEGFR‐2 (vascular endothelial growth factor receptor 2; AlexaFluor 555, red) and actin (AlexaFluor 488, green), VE‐cadherin (vascular endothelial cadherin; AlexaFluor 555, red), eNOS (endothelial nitric oxide synthase; AlexaFluor 555, red), PECAM‐1 (platelet endothelial cell adhesion molecule‐1; AlexaFluor 555, red), UEA‐1 (Ulex europaeus‐1 lectin; AlexaFluor 488, green), ac‐LDL uptake (acetylated low‐density lipoprotein; AlexaFluor 488, green), and progenitor cell surface marker CD34 (cluster of differentiation 34; AlexaFluor 488, green). Nuclei were stained with Topro‐3 iodide (blue) in confocal microscope magnification: ×63.

ECFCs were isolated from the peripheral blood of 11 participants. Fluorescent‐activated cell sorting analysis confirmed that >99% of the ECFCs were negatively stained for hematopoietic biomarker CD45 and positively stained for the endothelial biomarkers PECAM‐1 (CD31), and VEGFR‐2 (Figure S2). These findings were further confirmed by immunofluorescence staining of ECFCs, HUVECs (an endothelial cell positive control), and human dermal fibroblast (negative control) against common endothelial surface biomarkers including VEGFR‐2, VE‐cadherin, eNOS, PECAM‐1 (CD31), UEA‐1 (Ulex europaeus agglutinin‐1), and AcLDL uptake (Figure 1A through 1C). ECFCs also stained positive for progenitor biomarker CD34 (cluster of differentiation 34). Phase‐contrast images also showed that ECFCs and HUVECs similarly exhibited cobblestone‐patterned endothelial‐like morphology.

### Functional and Dysfunctional ECFCs

**Figure 2:**
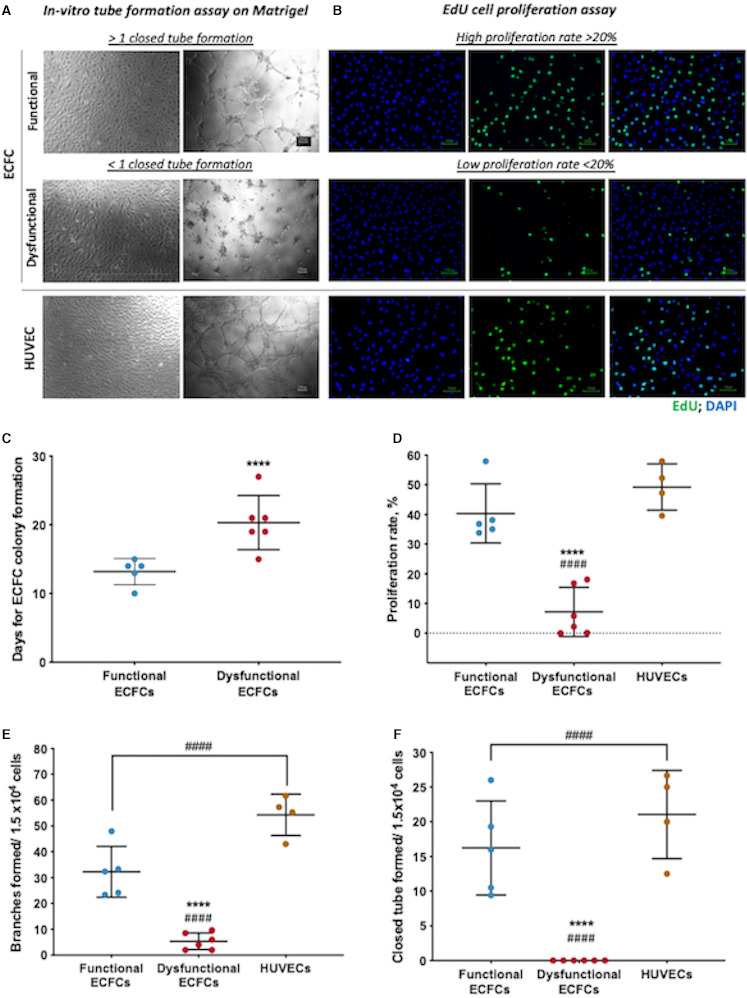
Functionality of endothelial colony‐forming cells (ECFCs). **A**, ECFC (functional and dysfunctional) and human umbilical vein endothelial cells (HUVECs) (positive endothelial control) tube formation capacity were assessed using Matrigel tube formation assay with the numbers of total branches and closed tubes formed quantified in phase‐contrast images (×5 objective). ECFCs with <1 closed tube formed were considered to be dysfunctional as depicted in the phase contrast images. **B**, Proliferation rate was determined by DNA Edu incorporation (AlexaFluor 488, green), with nuclei counterstained with 4′,6‐diamidino‐2‐phenylindole (DAPI; blue) (×10 objective). Dysfunctional cells and functional cells were determined with a proliferation rate (% Edu‐stained cells/DAPI stained ×100) of below 20% and above 20%, respectively. Scatter plots representing respective ECFC clusters and HUVECs for each of the following cell characteristics: (**C**) days for ECFC colony formation, (**D**) proliferation rate (%), (**E**) total branching formed per 1.5×10^4^ cells, and (**F**) total closed tubes formed per 1.5×10^4^ cells. Functional ECFCs (n=5), dysfunctional ECFCs (n=6), and HUVECs (n=4). ****P<0.0001 vs cluster 1 and ^####^ *P*<0.0001 vs HUVECs. Mann‐Whitney *U* test (**C**) or Kruskal‐Wallis test with Bonferroni post hoc test (**D** through **F**).

The comparison of ECFC functional characteristics revealed that, in cells of subjects with dysfunctional ECFCs, the number of days for ECFC colony formation was longer (20.3±3.0 versus 13.2±1.9 days, *P*<0.001), the proliferation rate was lower (7.2%±8.2% versus 40.3%±10%, *P*<0.001), and the angiogenic capacity was reduced as indicated by the number of tube branching (5.4±3.3 versus 32.3±9.9 branches, *P*<0.001) and the number of closed tubes formed in vitro (0 versus 16.2±6.8 closed tubes, *P*<0.001) (Figure 2A through 2F). When comparing ECFCs to HUVECs, we found that functional ECFCs displayed a similar proliferation rate to HUVECs (40.3%±10.0% versus 49.3%±7.8% respectively), although they had lower angiogenic capacity demonstrated by reduced branching (32.3±9.9 versus 54.3±8.0 branches, *P*<0.001) and the number of closed tubes (16.2±6.8 versus 21.0±6.4 closed tubes, *P*<0.001) (Figure 2D through 2F).

A comparative proteomic analysis between overall ECFCs with HUVECs further confirmed a similar proteomic profile between these 2 types of endothelial cells (average 97.9% of matched proteins). The global protein expression profile (presented as ECFC/HUVEC log2protein expression fold change) revealed that both ECFCs and HUVECs express endothelial lineages NRP‐2 (neuropilin‐2, −1.1), CD34 (−2.0), vWF (von Willebrand factor, 1.8), PECAM‐1 (−0.04), VEGFR‐2 (2.6), ECSCR (endothelial cell‐specific chemotaxis receptor, 1.0), cadherin 5 (CDH5, 0.14), ERG (ETS‐related gene, 2.5), and the extracellular matrix and basement membrane proteins (COL1A1 [collagen, type I, alpha 1], COL1A2 [collagen, type I, alpha 2], COL4A1 [collagen type IV alpha 1], COL4A2 [collagen type IV alpha 2]). Based on the proteomic profile, some proteins are exclusively expressed in ECFCs while undetectable in HUVECs, which include proteins involved in protein modification including ALG1 (chitobiosyldiphosphodolichol β‐mannosyltransferase), AKAP11 (A‐kinase anchor protein 11), NDST2 (N‐sulfotransferase 2), PKN3 (serine/threonine‐protein kinase N3), SETD3 (histone‐lysine N‐methyltransferase), and extracellular matrix organization protein NID‐2 (nidogen‐2). In addition, our proteomic analyses did not detect a CD133 protein amount either in ECFCs or in HUVECs. Hierarchical clustering comparing ECFCs and HUVECs revealed that functional ECFCs exhibited a greater proteome similarity with HUVECs (99.1%±0.16% matched pairs, Pearson correlation=0.77±0.07, *R* ^2^=0.59±0.11) than dysfunctional ECFCs (96.9%±1.27% matched pairs, Pearson correlation=0.54±0.04, *R* ^2^=0.30±0.04) (Figure S4).

### Clinical Characteristics of Subjects With Functional and Dysfunctional ECFCs

Clinical characteristics of the 11 participants are presented in Table 1. No differences were observed for male/female numbers, age, height, and weight between functional versus dysfunctional ECFC groups. However, systolic and mean blood pressures, as well as BMI, were significantly higher in subjects with dysfunctional ECFCs. Diastolic blood pressure and heart rate were similar between the groups (Table 1). In addition, differences in blood lipid profiles were observed, with higher total cholesterol and low‐density lipoprotein in subjects with dysfunctional ECFCs compared with subjects with functional ECFCs, with no significant differences observed for triglycerides, high‐density lipoprotein, glucose, insulin, homeostasis model assessment β‐cell, homeostasis model assessment β‐cell sensitivity, and homeostasis model assessment insulin resistance between groups (Table 1).

**Table 1:** Clinical Characteristics of the Study Participants

	Total, n=11	Functional ECFC, n=5	Dysfunctional ECFC, n=6	*P* value
Clinical characteristics				
Men, n (%)	5 (45)	2 (40)	3 (50)	>0.99
Age, y	26.5±4.6	24.8±1.6	28.0±5.9	0.245
Height, cm	171.6±4.6	173.8±10.7	169.8±7.2	0.482
Weight, kg	70.9±11.9	64.0±7.8	76.7±12.1	0.074
BMI, kg/m^2^	24.1±4.1	21.2±1.8	26.6±3.8	0.016*
Systolic blood pressure, mm Hg	129.7±14.0	120.9±8.3	137.1±14.0	0.050*
Diastolic blood pressure, mm Hg	83.0±12.2	75.5±13.7	89.3±6.7	0.089
Mean arterial pressure, mm Hg	100.6±13.0	91.8±11.2	107.9±11.4	0.032*
Heart rate, bpm	72.3±10.7	71.3±11.0	73.2±11.4	0.789
Blood biochemistry				
Triglyceride, mmol/L	1.1±0.8	0.9±0.3	1.4±1.0	0.328
Cholesterol, mmol/L	4.8±0.8	4.2±0.6	5.3±0.4	0.004*
High‐density lipoprotein, mmol/L	1.4±0.3	1.4±0.2	1.4±0.3	0.838
Low‐density lipoprotein, mmol/L	2.6±0.4	2.1±0.5	3.1±0.7	0.027*
Glucose, mmol/L	4.9±0.4	4.8±0.3	5.0±0.5	0.433
Insulin, pmol/L	49.8±14.1	45.9±10.4	53.0±16.7	0.433
HOMA β cell, %	96.4±24.3	95.4±19.3	97.3±29.7	0.904
HOMA β‐cell sensitivity, %	116.4±33.4	123.1±31.3	110.7±37.0	0.569

Data are expressed as mean±standard deviation. BMI indicates body mass index; ECFC, endothelial colony‐forming cell; and HOMA, homeostasis model assessment.

* *P*<0.05 between functional clusters, independent *t* test or χ^2^ test.

### Proteomic Analysis of Functional and Dysfunctional ECFCs

**Figure 3:**
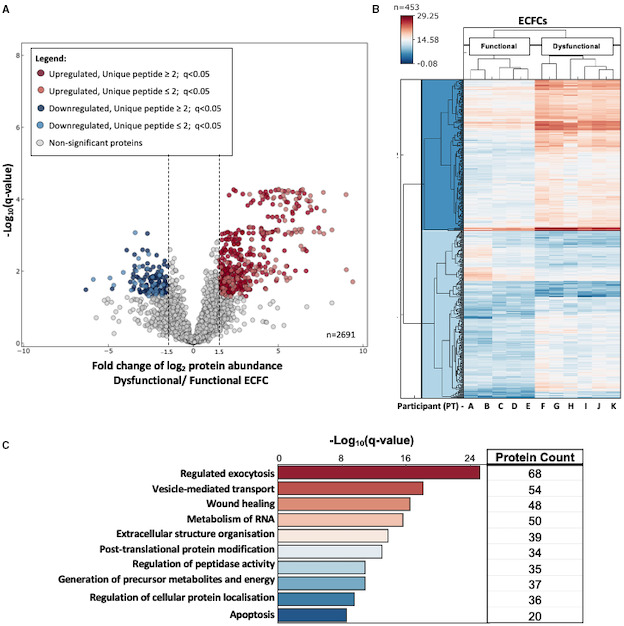
Proteomics analysis of functional and dysfunctional endothelial colony‐forming cells (ECFCs). **A**, Volcano plot shows significantly regulated proteins between dysfunctional and functional ECFCs, after Student *t* test and false discovery rate correction for multiple testing. A total of 2691 proteins were quantified with 453 proteins demonstrating significant differential abundance between the compared conditions based on proteins with unique peptides ≥2 and Log2 (fold change) ≥1.5 or ≤−1.5 (357 upregulated and 96 downregulated proteins). **B**, Unsupervised hierarchical clustering (Euclidean distance) distinguishes samples according to their group and shows distinct protein abundance profiles indicative of grouping. **C**, Significantly changed proteins show enrichment gene ontology biological pathways. Protein count represents the number of proteins involved in the respective biological pathway.

Proteomic comparison of ECFCs further identified 2691 proteins with 519 differentially expressed proteins between functional and dysfunctional clusters with unique peptides ≥2 and either Log_2_(fold change) ≥1.5 or ≤−1.5 (388 upregulated and 131 downregulated proteins) (Figure 3A and 3B). The analysis of gene ontology revealed the top 10 enriched pathways being the following: regulated exocytosis, vesicle‐mediated transport, metabolism of RNA, wound healing, extracellular matrix organization, generation of precursor metabolites and energy, posttranslational protein modification, apoptosis, regulation of peptidase activity, and regulation of cellular protein localization (Figure 3C and Figure S5).

**Figure 4:**
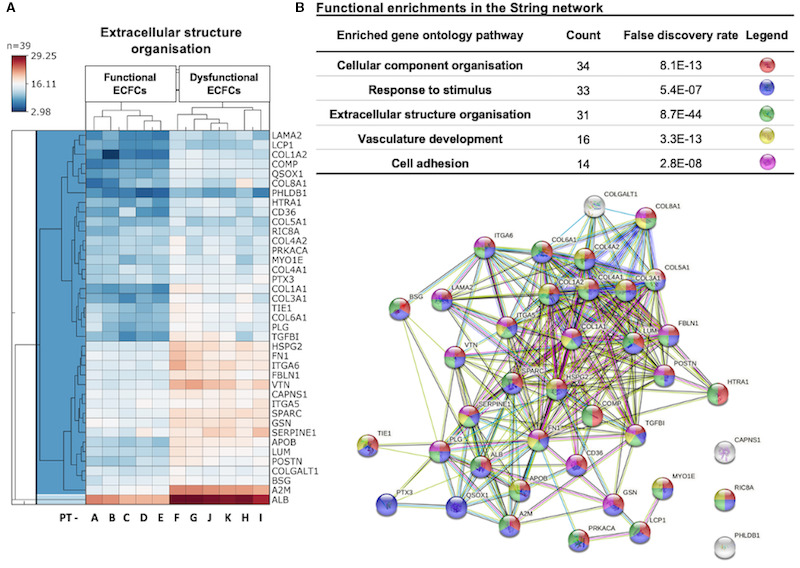
Proteins identified in enriched gene ontology pathway–extracellular structure organization that differently expressed between functional clusters of endothelial colony‐forming cells (ECFCs). **A**, Heatmap of proteins involved in the extracellular structure organization biological pathway. Results are represented as a heatmap displaying protein expression levels on a logarithmic scale. Red indicates high expression, whereas dark blue indicates low or no expression. **B**, String interactive pathway analysis of proteins involved in extracellular structure organization (n=39).

Detailed proteomic investigation of the impaired functionality in dysfunctional ECFCs revealed significant differences in 42 proteins involved in the extracellular structural organization (Figure 4A and 4B). Within the identified enriched gene ontology pathway, 4 proteins of interest have been identified. These proteins include SPARC (secreted protein acidic and cysteine rich), also known as OSN (osteonectin), PTX3 (pentraxin‐related protein PYX3), LUM (lumican ), and CD36 (cluster of differentiation 36) (Table 2). However, after adjusting for BMI and systolic blood pressure, only SPARC and LUM remained significantly different between functional and dysfunctional ECFCs (Table 2).

**Table 2:** Identified Proteins of Interest From the Extracellular Structure Organization (Gene Ontology Pathway)

Protein name	Functional description	Functional ECFCs log_2_protein expression±SD	Dysfunctional ECFCs log_2_protein expression±SD	Dysfunctional/functional log_2_protein expression fold change	Unique peptide count	−Log_10_ *P* value*	*P* value^†^, ^‡^
SPARC (secreted protein acidic and cysteine rich)	Antiangiogenesis; antiproliferation; antiadhesion; regulates endothelial cell migration, VEGFR signaling, ECM structural constituent, remodeling, collagen and calcium binding	15.0±0.5	18.2±0.5	3.21	12	6.14	0.0003
PTX3 (pentraxin‐related protein PYX3)	Antiangiogenesis; inhibits vascular regeneration; regulates ECM remodeling, inflammation, nitric oxide biosynthesis, and phagocytosis	12.3.5±0.9	14.9±0.7	2.64	8	3.34	0.065
LUM (lumican)	Antiangiogenesis; regulates cartilage development, ECM organization, keratan sulfate biosynthesis, and catabolic processes; RNA transcription; and positive regulation of transforming growth factor‐β1 production	12.4±0.4	16.8±0.8	4.38	3	5.65	<0.0001
CD36 (cluster of differentiation 36)	Antiangiogenesis, regulates cell adhesion, inflammatory response, fatty acid metabolism, cholesterol metabolism, and platelet degranulation	9.6±2.2	13.3±1.1	3.70	2	2.25	0.13

ECFCs indicates endothelial colony‐forming cells; ECM, extracellular matrix; and VEGFR, vascular endothelial growth factor receptor.

* Independent *t* test comparison after correction for false discovery rate by applying the Benjamini and Hochberg algorithm.

^†^ Independent *t* test.

^‡^ *P* value corrected for body mass index and systolic blood pressure.

## Discussion

This study is the first to describe a distinct proteomic signature and to target altered angiogenic mechanisms in dysfunctional ECFCs with significant reduction in both expansion and vasculogenic capacities. In addition, we also provide evidence that specific proteomic changes in dysfunctional cells were associated with a distinct ECFCs phenotype and molecular profile in comparison with another highly angiogenic endothelial cell population of HUVECs.

To properly run such comparisons, our study performed a comprehensive characterization of ECFCs showing the expression of classic endothelial cell surface markers including PECAM‐1 (CD31), VE‐cadherin, VEGFR‐2, eNOS, lectin (UEA‐1), but also key progenitor cell markers such as AcLDL and CD34 in ECFCs. These findings are in line with others supporting the use of a combination of PECAM‐1/VE‐cadherin/VEGFR‐2–positive and CD45‐negative surface markers when quantifying ECFCs from blood or PBMC pools by flow cytometry.^12^, ^23^, ^26^ In addition, our study confirmed the expression of CD34, a marker of progenitor cells in ECFCs.

A proteome comparative analysis revealed similar proteomic profiles between overall ECFCs and HUVECs, although this degree of similarity varied according to ECFC functionality. These data are consistent with recent findings from Kutikhin et al that showed similar proteome profiles between ECFCs and HUVECs, particularly in the expression of specific endothelial lineage, extracellular matrix, and basement membrane markers including NRP‐2, PECAM‐1, COL1A1, COL1A2, COL4A1, and COL4A2.^37^ In contrast to our findings, the authors demonstrated higher proliferative potential in ECFCs than HUVECs.^37^ This may be because of differences in baseline clinical characteristics of the study population or to sex differences, because they assessed ECFCs obtained only from male subjects.

Proteomic studies have been conducted to define molecular mechanisms and markers in cardiovascular diseases and regeneration, both experimentally and clinically. Proteins involved in angiogenesis, vasoconstriction, inflammation, and matrix degeneration have been repeatedly found in such studies.^38^ However, to this date, the main mechanisms impairing angiogenesis in primary cultured ECFCs are not entirely elucidated. Therefore, our proteomic findings are among the first to identify mechanisms significantly altered in less angiogenic and proliferative ECFCs. The gene ontology of distinct proteomic signatures between functional clusters revealed the activation of mechanisms driving exocytosis regulation and vesicle‐mediated transport processes in dysfunctional ECFCs, which are critical processes involved in the formation and secretion of micro‐ and nanovesicles in endothelial cells.^39^ In response to injury and stress, endothelial cells can undergo exocytosis by releasing numerous hemostatic and proinflammatory factors into the blood stream, which can further regulate vascular thrombosis and inflammatory responses.^40^ One of the main factors released by endothelial cells is the prothrombotic protein vWF.^40^, ^41^ Some studies have shown that patients with increased vWF levels have a higher incidence of adverse cardiovascular events including coronary artery disease, myocardial infarction, and thromosis.^42^, ^43^ In addition, animal studies have also highlighted the relationship of exocytosis and endothelia dysfunction. A study by Zhang et al in mice outlined the impact of excessive exocytosis of lysosomal‐related organelles, which hindered endothelial mechano‐transduction and nitric oxide production, leading to endothelial cell dysfunction.^44^

Furthermore, our proteomic analysis also identified changes in mechanisms essential for maintaining the vascular tree, including extracellular matrix organization and regulation of apoptosis.^45^, ^46^, ^47^ These biological functions are also critical for capillary sprouting and wound healing.^48^ In addition, we identified changes in main mechanisms regulating RNA metabolism, as well as the generation of precursor metabolites and energy in cells, indicating significant metabolic and energetic changes in dysfunctional ECFCs.

We also identified targeted proteins that were differently expressed between functional clusters of ECFCs. Top‐ranked proteins upregulated in the group of dysfunctional ECFCs were SPARC, CD36, LUM, and PTX3. It is beyond the scope of this study to review the functions of these proteins extensively, but these are briefly described in Table 2. Interestingly, CD36 and SPARC are cellular receptors to thrombospondin‐1, an essential antiangiogenic focal adhesion glycoprotein with cellular effects that can inhibit cell migration and tube formation in endothelial cells.^49^, ^50^ In addition, SPARC is shown to reduce endothelial cell proliferation mainly by antagonizing vascular endothelial growth factor and inhibiting the phosphorylation of mitogen‑activated protein kinases and fibroblast growth factor.^51^

Similarly, LUM is a small leucine‐rich proteoglycan that binds to the α2 integrin I domain and significantly reduces the expression of matrix metalloproteinases, particularly matrix metalloproteinase‐14, inhibiting cell adhesion and migration in endothelial cells.^52^ Furthermore, LUM is a novel mediator of angiostasis by promoting fibrillogenesis and the stabilization of collagen fibers. It is also reported to inhibit tumor angiogenesis and growth by activating the proapoptotic Fas pathway in endothelial cells.^53^

Interestingly, after adjusting the top‐ranked proteins by the participants' BMI and systolic blood pressure, we found that only SPARC and LUM remained significantly different between functional and dysfunctional ECFCs, whereas PTX3 and CD36 were no longer significantly different. These findings suggest a closer relationship between the latter 2 proteins with BMI and systolic blood pressure variations. PTX3 is a soluble pattern‐recognition receptor, produced mainly by monocytes and endothelial cells. It binds with high affinity to FGF2 (fibroblast growth factor‐2) and heparan sulfate proteoglycans, thus inhibiting angiogenesis and cell proliferation.^54^, ^55^ Elevated PTX3 levels were also reported in various cardiovascular diseases including pulmonary arterial hypertension,^56^ acute coronary syndrome,^57^ heart failure,^58^ and in advanced atherosclerotic plaques.^59^

Our findings and others' suggest that the disruption of these candidate antiangiogenic proteins and mechanisms could contribute to the progression of cardiovascular diseases in subjects.^51^, ^52^, ^53^, ^54^, ^55^, ^56^, ^57^, ^58^ Emerging evidence suggests that ECFCs play an active role in stimulating processes of capillary sprouting and angiogenesis during wound healing and tissue development, as well as by contributing to the maintenance and remodeling of main vessels in physiological processes during pregnancy and diseases such as hypertension.^4^, ^5^, ^11^, ^27^, ^28^, ^60^, ^61^, ^62^, ^63^ Deficiency in number and activity of circulating overall EPCs and ECFCs were previously associated with reduced arterial elasticity in humans with advancing ageing and common cardiovascular risk factors like diabetes mellitus, hypertension, heart failure, ischemic stroke, angina, atherosclerotic coronary artery lesions, and hyperlipidemia.^27^, ^64^, ^65^, ^66^, ^67^, ^68^ In addition, the strong association between ECFC dysfunction and higher blood pressure was also reported in preterm‐born young adults, particularly in those exposed to bronchopulmonary dysplasia as newborns, which is a comorbidity marked by severe microvascular growth arrest in newborn lungs.^6^, ^16^ These studies corroborate to our findings showing impaired proliferative and angiogenesis potentials in subjects with dysfunctional ECFCs also presenting higher blood pressure values. ECFC dysfunction could, therefore, represent a pivotal mechanism to the initiation of the pathogenesis of cardiovascular diseases with associated endothelial dysfunction in these subjects.

Furthermore, higher BMI was also observed in participants with dysfunctional ECFCs. This finding was also evident in studies conducted by MacEneaney et al and Tobler et al that revealed a reduction in circulating overall EPCs and ECFCs, with premature cell senescence and impaired ECFC colony‐forming and proliferative capacity in overweight and obese adults subjects.^69^, ^70^ A reduction in EPC number in obese individuals was also evident in several studies, including patients with obesity‐related hypercholesterolemia.^71^, ^72^ The mechanisms promoting EPC, and particularly ECFC, dysfunction in obesity and dyslipidemia is still unclear. However, it may involve the increase in adiposity observed in these individuals, which could prevent EPCs from releasing proangiogenic factors including VEGF and granulocyte colony‐stimulating factor while promoting the release of proapoptotic caspase‐3, thereby compromising their reparative potential.^73^

Despite a limited study sample size, our investigations are in line with others suggesting a close link between underlying cardiovascular disease risks and endothelial cell dysfunction. Several studies have observed sex‐specific differences in the intracellular proteome and genome of human endothelial cells, which may affect endothelial cell function.^74^, ^75^, ^76^, ^77^ Because of our limited sample size, we were unable to study the effect of sex on ECFC function and proteome profiles. Sex‐based ECFC differences are still unknown. Additional studies are needed to confirm and validate the main observations and mechanisms, establishing a connection between our experimental findings with clinical characteristics, taking into account potential sex differences. Additionally, this proteomic approach has successfully identified a novel ECFC proteomic signature and targeted proteins potentially driving ECFC angiogenic (dys)function.

## Conclusions

Circulating ECFCs with impaired angiogenesis and expansion capacities have distinct proteomic profile and phenotype in comparison with highly angiogenic ECFCs and HUVECs. The disruption of key cellular mechanisms involved mainly in exocytosis and vesicle transport as well as in extracellular matrix organization, cell metabolism, and apoptosis were identified in dysfunctional ECFCs, in addition to targeted antiangiogenic proteins. These findings suggest a potential relationship between specific antiangiogenic mechanisms and endothelial cell dysfunction. However, more studies are needed to validate our targeted mechanisms in larger populations to enhance our understanding of the physiological function of ECFCs and their potential clinical applications in cardiovascular diseases.

## Sources of Funding

Study funding was provided by Wellcome Trust (grant no. 105741/Z/14/Z), the British Heart Foundation (grant no. PG/17/13/32860), and the Oxford British Heart Foundation Centre of Research Excellence (grant no. RE/13/1/30181). Dr Lewandowski is funded by a British Heart Foundation Intermediate Basic Science Research Fellowship (grant no. FS/18/3/33292). Dr Bertagnolli is funded by grants from CIUSSS Nord‐de‐l'Île‐de‐Montréal, SickKids Foundation, and Canadian Institutes of Health Research (grant no. NI20‐1037).

## Disclosures

None.

## Supporting information

Table S1
Figures S1–S5
