# Supplementary material for: Proteomic Signature of Dysfunctional Circulating Endothelial Colony‐Forming Cells of Young Adults
Source: J Am Heart Assoc. 2021 Jul 19;10(15):e021119. doi: 10.1161/JAHA.121.021119 (PMC8475699; doi:10.1161/JAHA.121.021119)
Supplement: Supplementary file 1 — Table S1 Figures S1–S5 [file JAH3-10-e021119-s001.pdf]

SUPPLEMENTAL MATERIAL

Table S1. List of antibodies used with the respective application.

Antigen	Spices	Dilution	Supplier	Application
**PECAM-1, PAb**	Rabbit	1:200	Thermo-Fisher, PA5-14372	IF
**CD34, MAb**	Mouse	1:200	Thermo Fisher, MA1-10202	IF
**eNOS, PAb**	Rabbit	1:200	Thermo Fisher, PA5-16887	IF
**VE-Cadherin, MAb**	Rabbit	1:200	Cell signalling technology, 2500S	IF
**Lectin (UEA-1) Fit-C conjugate**	-	1:200	Vector laboratories, B-1065	IF
**VEGF-R2, MAb**	Rabbit	1:200	Stratech Scientific Ltd, 50998-R004-P	IF
**TO-PRO™-3 Iodide (642/661)**	-	1:500	Invitrogen, T3605	IF
**ActinGreen™ 488 ready probes™ reagent**	-	1 drop/ 0.5ml	Thermo Fisher, R37110	IF
**AlexaFlour™ 555 anti-rabbit IgG(H+L)**	Goat	1:400	Invitrogen, A27039	IF, Flow cytometry
**AlexaFlour™ 488 anti-mouse IgG**	Goat	1:400	Invitrogen, A1101	IF, Flow cytometry
**Human VE-Cadherin (D87F2) XP® PE Conjugate, MAb**	Rabbit	1: 150	Cell Signalling Technology, 89426S	Flow cytometry

**Human PECAM-1 Fit-C conjugate, MAb**	Mouse	1:20	Bio Legend Inc, 303104	Flow cytometry
**Human VEGF R2 (Clone 89106), MAb**	Mouse	1:20	Bio-Techne (R&D Systems); FAB357A	Flow cytometry
**Mouse IgG1 kappa Isotype Control**	Mouse	1:200	Invitrogen; 14-471482	IF
**Rabbit IgG Isotype Control**	Rabbit	1:200	Life technologies, 086199	IF

Polyclonal antibody (PAb); Monoclonal antibody (MAb); Immunofluorescence staining (IF)

Figure S1. Schematic representation of endothelial colony-forming cells (ECFCs) isolation and colony formation.

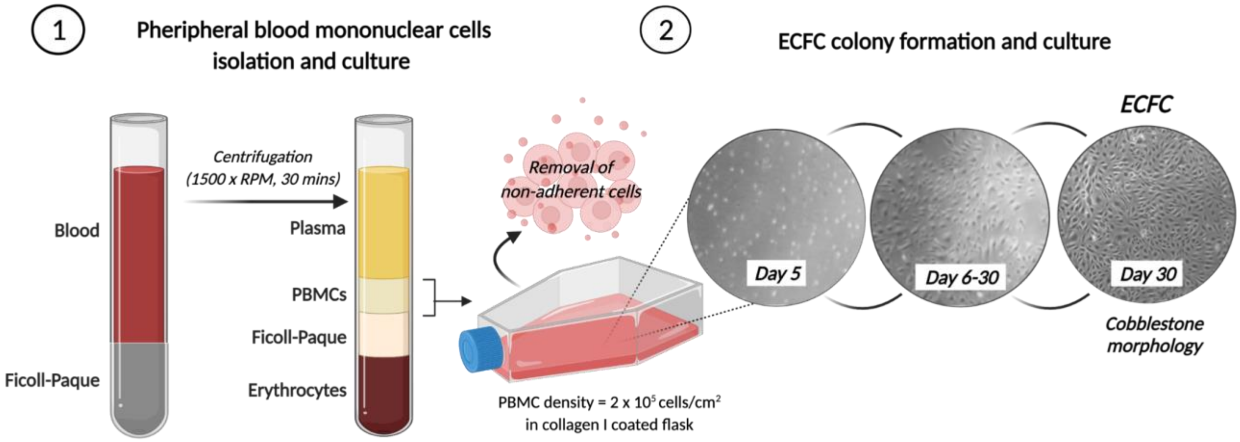

Peripheral blood mononuclear cells (PBMCs) isolated from peripheral blood undergo cell culture and were observed daily from day 6 to 30 to determine the first day of cobblestone-pattern ECFC colony formation (phase contrast fluorescence microscope, magnification 5x).

Figure S2. Fluorescence-activated cell sorting (FACS) of endothelial colony-forming cells (ECFCs) represented in dot plots and histograms.

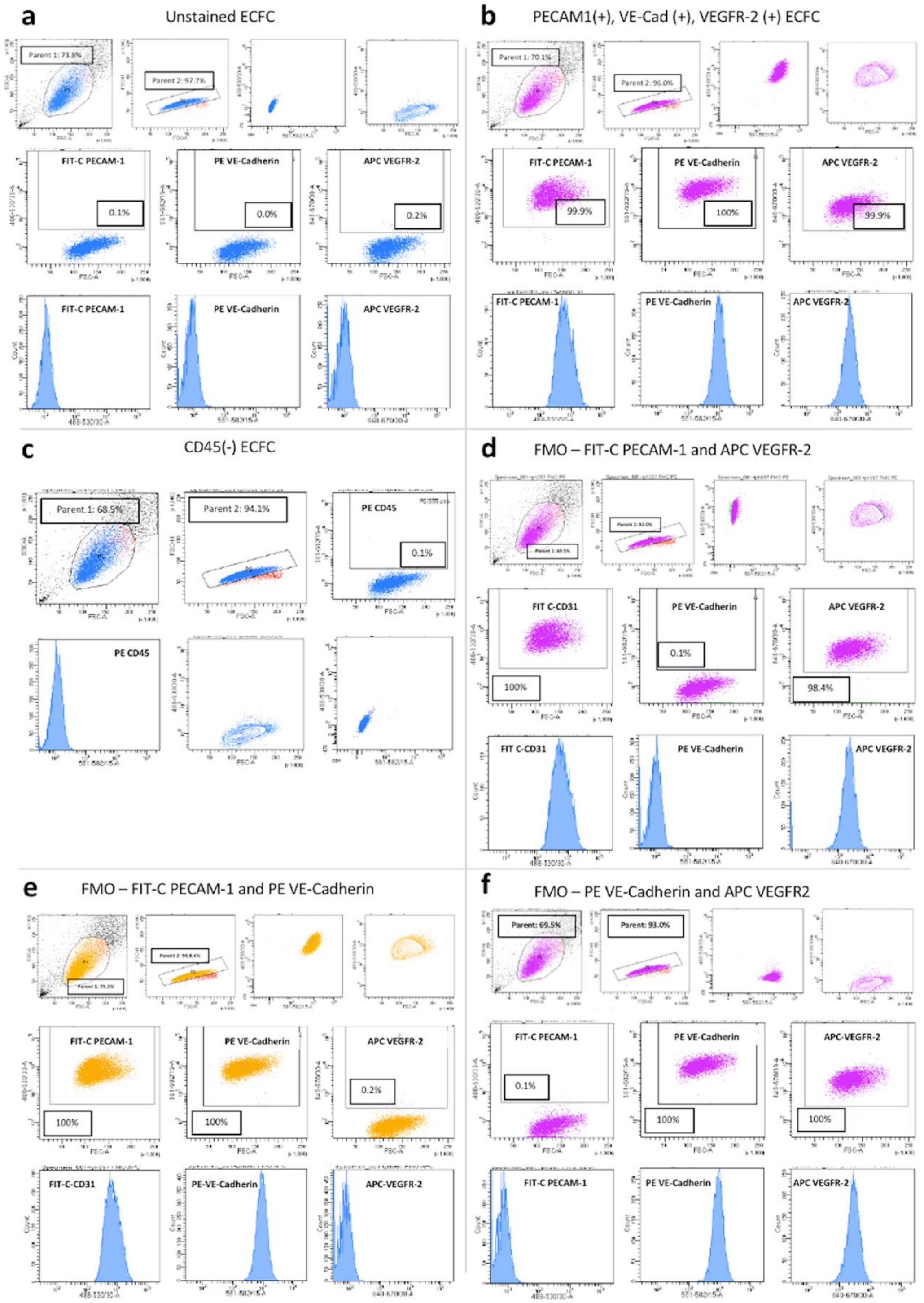

(a) Unstained ECFCs. **(b)** Cells were stained with endothelial cell surface biomarkers including FITC-PECAM-1, PE-VE-Cadherin, APC-VEGFR-2 and **(c)** with a hematopoietic cell lineage surface marker PE-CD45. FACS immunophenotype showed that ECFC expressed PECAM-1, VE-Cadherin, VEGFR-2, but were negative for CD45. The overall percentage is based on *Parent 2*. Fluorescence minus one controls (FMO) for ECFCs represented in dot plots and histograms. Cells were stained with **(d)** FIT-C PECAM-1 and APC VEGFR-2; **(e)** FIT-C PECAM-1 and PE VE-Cadherin; and **(f)** PE VE-Cadherin and APC-VEGFR-2.

Figure S3 - Endothelial colony-forming cells (ECFC) phenotype in comparison to human umbilical vein endothelial cells (HUVECs) and dermal fibroblasts.

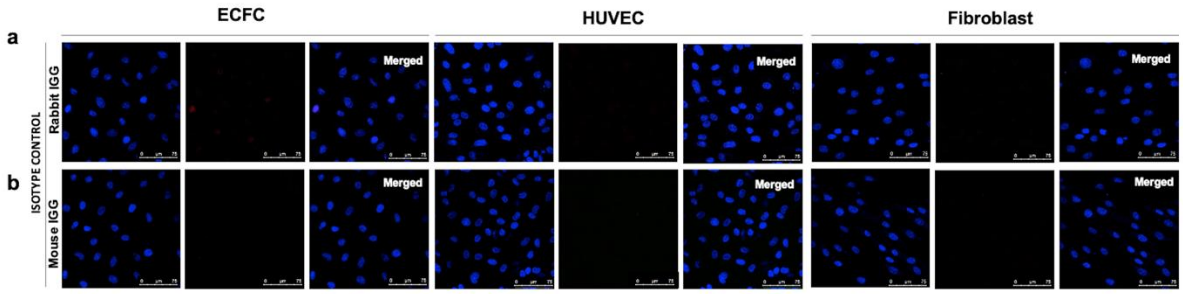

Immunoglobulin isotype controls including **(a)** mouse IGG (green) and **(b)** rabbit IGG control (red) were stained negative on all the cells. Nuclei were stained with Topro-3 iodide (blue) (confocal microscope magnification: 63x).

Figure S4. Proteomic comparisons and hierarchical clustering of endothelial colony-forming cells (ECFCs) versus human umbilical vein endothelial cells (HUVECs).

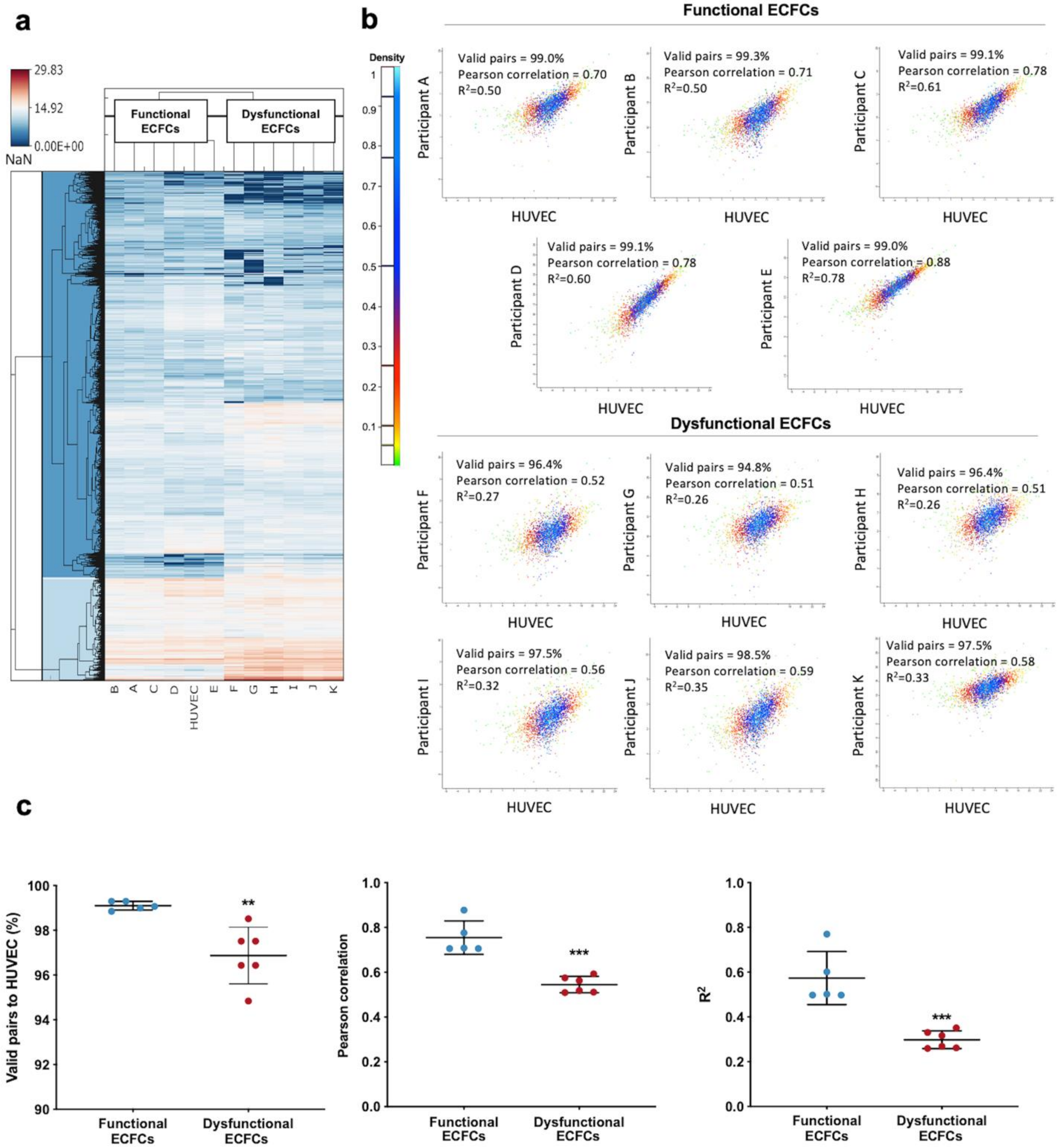

(a) A total of 2691 proteins were expressed among two distinct ECFC functional clusters. Results are represented as a heatmap displaying protein expression levels on a logarithmic scale. Red indicates high expression while dark blue indicates low or no expression. **(b)** Scatter plots of

normalized protein abundance in ECFCs against HUVECs. **(c)** Scattergram of % of valid matched protein pairs between ECFCs and HUVECs. Functional ECFCs displayed highly similar protein expression (%) compared to HUVECs (99.1±0.16%, Pearson correlation = 0.77±0.07, R2 = 0.59±0.11) while dysfunctional ECFCs had lower degree of similarity and correlation to HUVECs (96.9±1.27%, Pearson correlation = 0.54±0.04, R2 = 0.30±0.04). Mean±SD; **P<0.01, ***P<0.001.

Figure S5. Heat maps based on log2 transformed (protein abundance) of proteins in the top 10 enriched gene otology pathways.

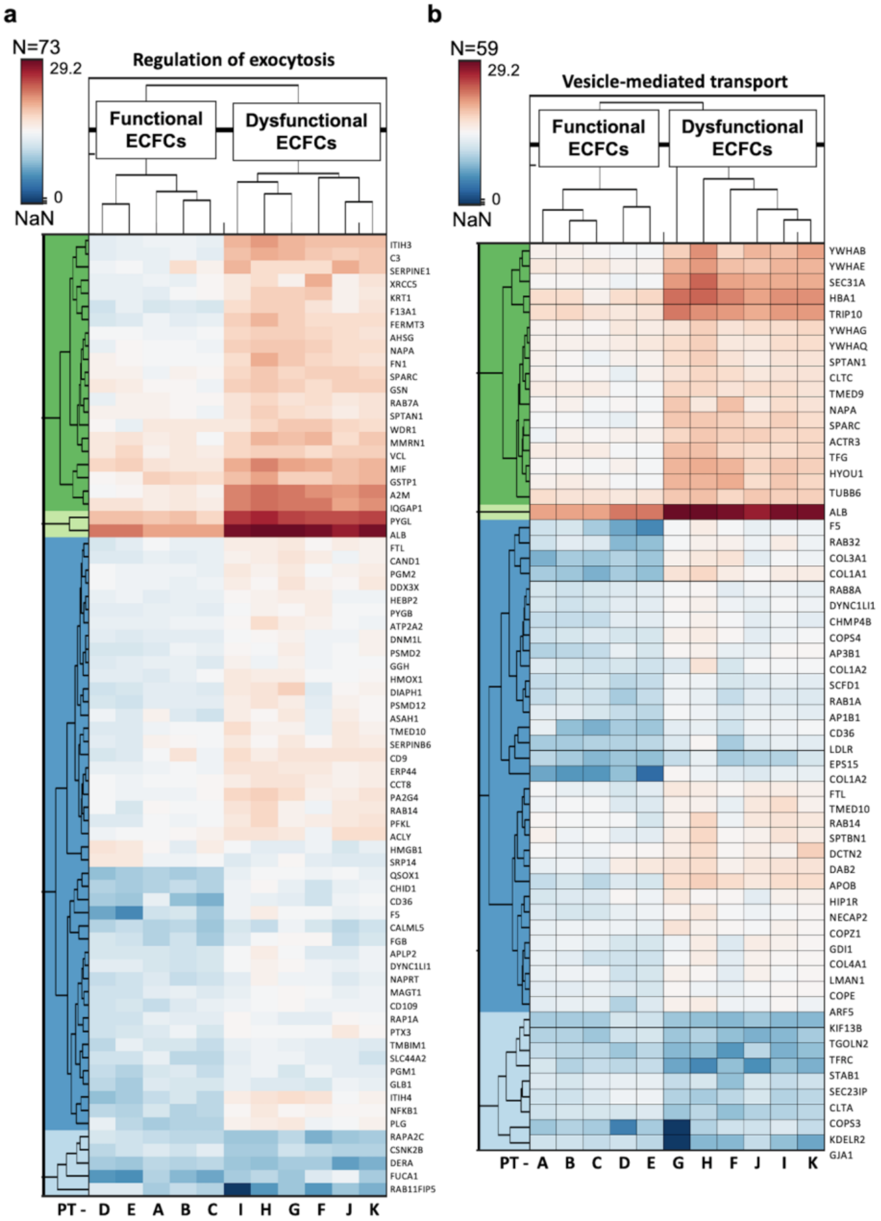

a
N=73
29.2
0
NaN
Regulation of exocytosis
Functional ECFCs
Dysfunctional ECFCs
ITIH3
C3
SERPINE1
XRCC5
KRT1
F13A1
FERMT3
AHSG
NAPA
FN1
SPARC
GSN
RAB7A
SPTAN1
WDR1
MMRN1
VCL
MIF
GSTP1
A2M
IQGAP1
PYGL
ALB
FTL
CAND1
PGM2
DDX3X
HEBP2
PYGB
ATP2A2
DNM1L
PSMD2
GGH
HMOX1
DIAPH1
PSMD12
ASAH1
TMED10
SERPINB6
CD9
ERP44
CCT8
PA2G4
RAB14
PFKL
ACLY
HMGB1
SRP14
QSOX1
CHID1
CD36
F5
CALML5
FGB
APLP2
DYNC1LI1
NAPRT
MAGT1
CD109
RAP1A
PTX3
TMBIM1
SLC44A2
PGM1
GLB1
ITIH4
NFKB1
PLG
RAPA2C
CSNK2B
DERA
FUCA1
RAB11FIP5
PT - D E A B C I H G F J K
b
N=59
29.2
0
NaN
Vesicle-mediated transport
Functional ECFCs
Dysfunctional ECFCs
YWHAB
YWHAE
SEC31A
HBA1
TRIP10
YWHAG
YWHAQ
SPTAN1
CLTC
TMED9
NAPA
SPARC
ACTR3
TFG
HYOU1
TUBB6
ALB
F5
RAB32
COL3A1
COL1A1
RAB8A
DYNC1LI1
CHMP4B
COPS4
AP3B1
COL1A2
SCFD1
RAB1A
AP1B1
CD36
LDLR
EPS15
COL1A2
FTL
TMED10
RAB14
SPTBN1
DCTN2
DAB2
APOB
HIP1R
NECAP2
COPZ1
GDI1
COL4A1
LMAN1
COPE
ARF5
KIF13B
TGOLN2
TFRC
STAB1
SEC23IP
CLTA
COPS3
KDELR2
GJA1
PT - A B C D E G H F J I K

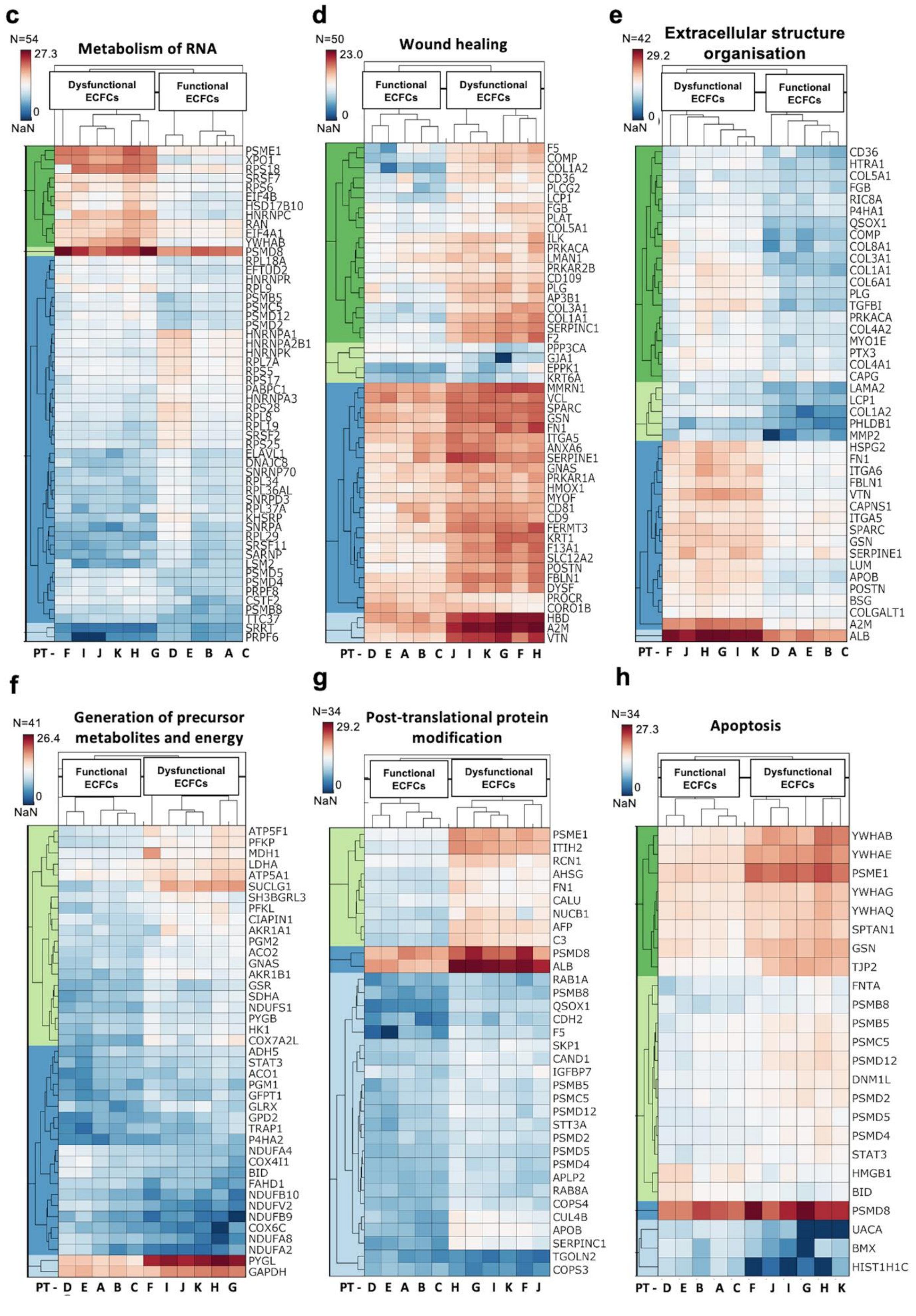

c
N=54
27.3
0
NaN
Metabolism of RNA
Dysfunctional ECFCs
Functional ECFCs
PSME1
XPO1
RPS18
SRSF7
RPS6
EIF4B
HSD17B10
HNRNPC
RAN
EIF4A1
YWHAB
PSMD8
RPL18A
EFTUD2
HNRNPR
RPL9
PSMB5
PSMC5
PSMD12
PSMD2
HNRNPA1
HNRNPA2B1
HNRNPK
RPL7A
RPS5
RPS17
PABPC1
HNRNPA3
RPS28
RPL8
RPL19
SRSF2
RPS25
ELAVL1
DNAJC8
SNRNP70
RPL34
RPL36AL
SNRPD3
RPL37A
KHSRP
SNRPA
RPL29
SRSF11
SARNP
LSM2
PSMD5
PSMD4
PRPF8
CSTF2
PSMB8
TTC37
SRRT
PRPF6
PT - F I J K H G D E B A C
d
N=50
23.0
0
NaN
Wound healing
Functional ECFCs
Dysfunctional ECFCs
F5
COMP
COL1A2
CD36
PLCG2
LCP1
FGB
PLAT
COL5A1
ILK
PRKACA
LMAN1
PRKAR2B
CD109
PLG
AP3B1
COL3A1
COL1A1
SERPINC1
F2
PPP3CA
GJA1
EPPK1
KRT6A
MMRN1
VCL
SPARC
GSN
FN1
ITGA5
ANXA6
SERPINE1
GNAS
PRKAR1A
HMOX1
MYOF
CD81
CD9
FERMT3
KRT1
F13A1
SLC12A2
POSTN
FBLN1
DYSF
PROCR
CORO1B
HBD
A2M
VTN
PT - D E A B C J I K G F H
e
N=42
29.2
0
NaN
Extracellular structure organisation
Dysfunctional ECFCs
Functional ECFCs
CD36
HTRA1
COL5A1
FGB
RIC8A
P4HA1
QSOX1
COMP
COL8A1
COL3A1
COL1A1
COL6A1
PLG
TGFBI
PRKACA
COL4A2
MYO1E
PTX3
COL4A1
CAPG
LAMA2
LCP1
COL1A2
PHLDB1
MMP2
HSPG2
FN1
ITGA6
FBLN1
VTN
CAPNS1
ITGA5
SPARC
GSN
SERPINE1
LUM
APOB
POSTN
BSG
COLGALT1
A2M
ALB
PT - F J H G I K D A E B C
f
N=41
26.4
0
NaN
Generation of precursor metabolites and energy
Functional ECFCs
Dysfunctional ECFCs
ATP5F1
PFKP
MDH1
LDHA
ATP5A1
SUCLG1
SH3BGRL3
PFKL
CIAPIN1
AKR1A1
PGM2
ACO2
GNAS
AKR1B1
GSR
SDHA
NDUFS1
PYGB
HK1
COX7A2L
ADH5
STAT3
ACO1
PGM1
GFPT1
GLRX
GPD2
TRAP1
P4HA2
NDUFA4
COX4I1
BID
FAHD1
NDUFB10
NDUFV2
NDUFB9
COX6C
NDUFA8
NDUFA2
PYGL
GAPDH
PT - D E A B C F I J K H G
g
N=34
29.2
0
NaN
Post-translational protein modification
Functional ECFCs
Dysfunctional ECFCs
PSME1
ITIH2
RCN1
AHSG
FN1
CALU
NUCB1
AFP
C3
PSMD8
ALB
RAB1A
PSMB8
QSOX1
CDH2
F5
SKP1
CAND1
IGFBP7
PSMB5
PSMC5
PSMD12
STT3A
PSMD2
PSMD5
PSMD4
APLP2
RAB8A
COPS4
CUL4B
APOB
SERPINC1
TGOLN2
COPS3
PT - D E A B C H G I K F J
h
N=34
27.3
0
NaN
Apoptosis
Functional ECFCs
Dysfunctional ECFCs
YWHAB
YWHAE
PSME1
YWHAG
YWHAQ
SPTAN1
GSN
TJP2
FNTA
PSMB8
PSMB5
PSMC5
PSMD12
DNM1L
PSMD2
PSMD5
PSMD4
STAT3
HMGB1
BID
PSMD8
UACA
BMX
HIST1H1C
PT - D E B A C F J I G H K

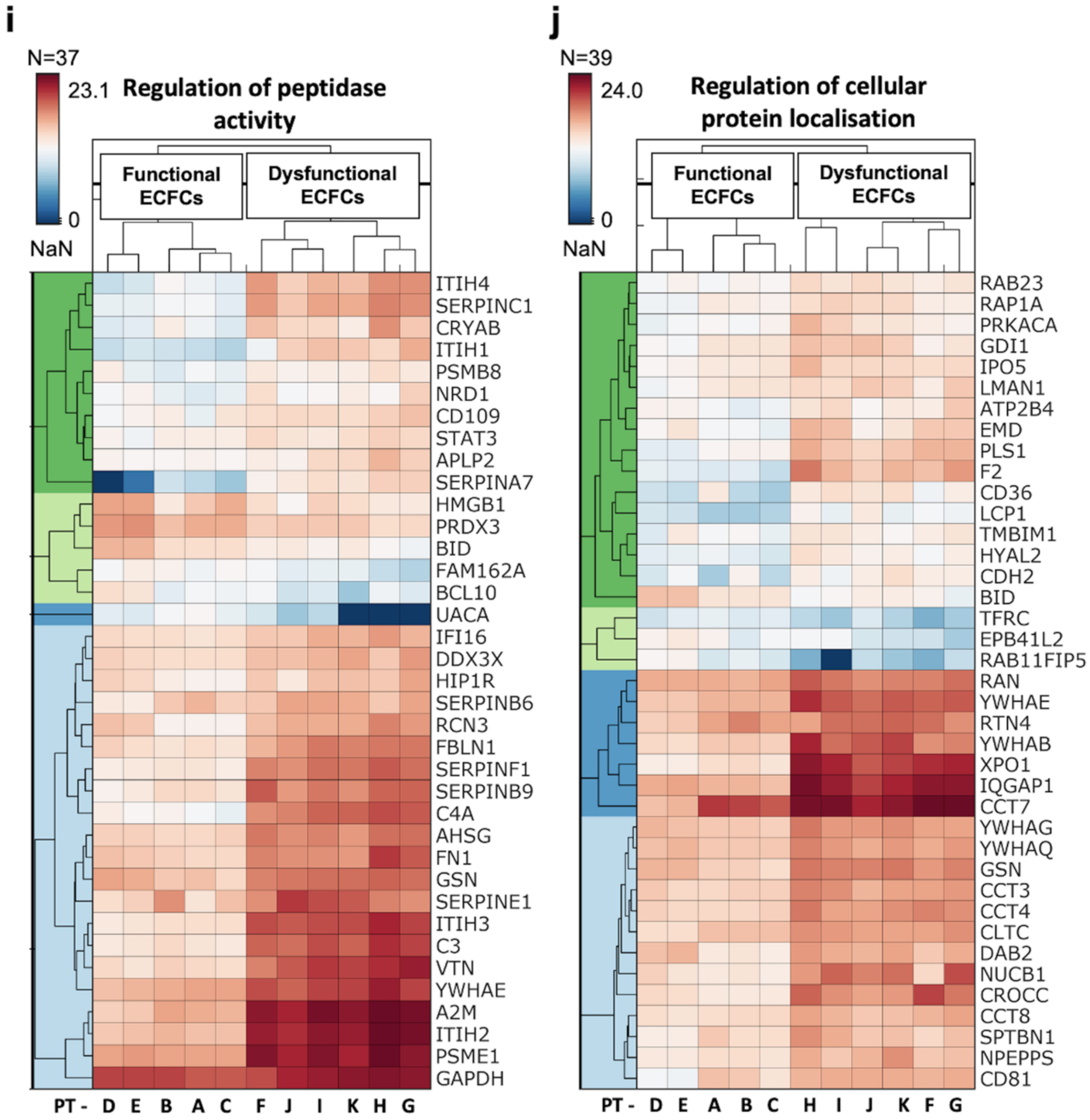

This includes **(a)** regulated exocytosis; **(b)** vesicle-mediated transport; **(c)** metabolism of RNA; **(d)** wound healing; **(e)** extracellular structure organization; **(f)** generation of precursor metabolites and energy; **(g)** post-translational protein modification; **(h)** apoptosis; **(i)** regulation of peptidase activity and **(j)** regulation of cellular protein localisation.
